# Gender differences in the clinical management of patients with angina pectoris: a cross-sectional survey in primary care

**DOI:** 10.1186/1472-6963-7-142

**Published:** 2007-09-04

**Authors:** Mike Crilly, Peter Bundred, Xiyuan Hu, Lisa Leckey, Fiona Johnstone

**Affiliations:** 1 Department of Public Health, University of Aberdeen Medical School, Polwarth Building at Foresterhill, Aberdeen, UK; 2 Department of Primary Care, University of Liverpool Medical School, Whelan Building, Liverpool, UK; 3 Liverpool Primary Care Trust, Newhall Campus, Longmoor Lane, Liverpool, UK; 4 Halton & St. Helens Primary Care Trust, Cowley Hill Lane, St. Helens, Merseyside, UK

## Abstract

**Background:**

Previous research suggests that women admitted to hospital with acute myocardial infarction (MI) are managed less intensively than men. Chronic stable angina is the commonest clinical manifestation of coronary heart disease in the community, but little information is available concerning its contemporary clinical management. The aim of this study is to assess the extent of gender differences in the clinical management of angina pectoris in primary care.

**Methods:**

A cross-sectional survey undertaken in 8 sentinel centres serving 63,724 individuals in the city of Liverpool (15% of the city population). Aspects of clinical care assessed included: risk factor recording (smoking, cholesterol, blood pressure, body mass index); secondary prevention (aspirin, beta-blocker, statin); cardiac investigation (exercise ECG, perfusion scanning, angiography); and revascularisation (percutaneous coronary intervention, coronary artery bypass grafting). Male-to-female adjusted odds ratios (AOR) were calculated (adjusted for age, angina duration, age at diagnosis and previous MI) using logistic regression.

**Results:**

1,162 patients (610 men; 552 women) with angina were identified. Women were older than men (71 vs 67 years), with a shorter duration of angina (6 vs 7 years), and a lower prevalence of previous MI (25% vs 43%). Men were significantly more likely than women to undergo detailed risk factor assessment (AOR = 1.35, 95%CI 1.06 to 1.73); receive 'triple' secondary prevention with aspirin, beta-blockers and statins (AOR = 1.47, 95%CI 1.07 to 2.02); access exercise ECG testing (AOR = 1.31, 95%CI 1.02 to 1.68); angiography (AOR = 1.61, 95%CI 1.23 to 2.12); and undergo coronary revascularisation (AOR = 1.93, 95%CI 1.39 to 2.68).

**Conclusion:**

Systematic gender differences exist in the comprehensive clinical management of patients with angina in primary care.

## Background

Previous research has demonstrated that important differences exist in the clinical management of men and women with suspected or established coronary heart disease (CHD) [1-7]. Most of this research has focused on the management of acute coronary syndrome (ACS) and the performance of revascularisation [1-6]. A recent systematic review of the diagnosis and treatment of CHD in women found considerable evidence that women admitted to hospital with ACS are less likely to receive aspirin, beta-blockers or thrombolysis; less likely undergo exercise stress testing; and also less likely to undergo angiography or revascularisation [8]. Although not all studies have found such gender differences, particularly after adjusting for important confounding factors such as age [9-13].

Most of the evidence for gender differences in the management of CHD relates to specialist care in the USA [1-5,8], although similar results have also been reported from hospitals in the UK [14-16]. Women presenting with angina appear to be at particular risk of receiving sub-optimal care in the hospital setting [4,17]. Despite the attention that has been focused on such gender differences over the past 20 years [1], such disparities seem to have remained largely unchanged in both the USA and UK over time [5,6].

Angina pectoris is the commonest clinical manifestation of CHD [18,19], but the management of chronic stable angina remains a neglected area of cardiovascular research [18]. Primary care physicians commonly manage angina in the community and may be predisposed to adopt a less favourable approach towards the clinical management of symptomatic women in both the USA and UK [20,21]. Few previous studies have examined gender differences in the primary care management of angina [22,23]. It is currently unclear as to what extent gender differences observed in hospital management are also present in the clinical care of patients with chronic stable angina in the community [8]. The aim of this study is to examine the influence of gender on the comprehensive clinical management of angina pectoris from a primary care perspective in the UK.

## Methods

This study reports a cross-sectional survey, undertaken by specially trained clinical data managers, of patients in primary care with an explicit and unequivocal physician diagnosis of angina pectoris.

### Sentinel primary care centres

Since 1992 several primary care centres across the city of Liverpool (sentinel practices) have employed a clinical data manager (CDM) as an additional member of their healthcare team. The CDMs have a remit for collecting anonymised individual patient data from primary care (as part of the Liverpool Primary Care Data Project) having undergone advanced training in data handling in the Department of Primary Care at Liverpool University.

Data collection was undertaken by 7 experienced CDMs (average of 6 years experience) attached to 8 sentinel practices (one CDM covered two practices). The sentinel practices served a combined registered population of 63,724 (15% of a city population of 439,473) and cover a range of geographical areas, from the most affluent to the most deprived parts of the city. The sentinel practices had participated in the primary care data collection project for between 6–9 years. All practices were computerised and ranged in size from 2,369 to 12,885 registered patients (median 8,307). Their combined age-sex profile was similar to the city as a whole, but with relatively fewer adults aged over 50 years compared with England (46% vs 54%). Information was extracted by the CDMs according to a standardised protocol. The quality of the data-collection process was overseen by an informatics co-ordinator. Data was collected using a controlled entry Microsoft Access Form within each practice. Data collection was piloted with 10 patients from each practice before the start of the study. The main study took 3-months (11 September to 18 December 2001). At the time of the study UK clinical guidelines did not recommend the use of ACE inhibitors [24] and statins were only available on prescription.

### Patients with angina

Patients prescribed any nitrate preparation over the previous 3 years (sub-lingual nitrates, tablets or patches as listed in the British National Formulary, BNF) were identified by searching the clinical computer systems. The CDMs reviewed both computerised and written medical records and extracted information only for those patients explicitly and unequivocally labelled with a physician diagnosis of angina pectoris. We considered such patients to have 'clinically certain angina' as described in evidence-based guidelines concerning the clinical management of chronic stable angina in UK primary care [24]. We excluded patients where there was any recorded doubt or disagreement concerning the diagnosis.

In addition to age, sex and the date of angina diagnosis, the CDMs also extracted information concerning the four major aspects of CHD care: (1) coronary risk factor recording (smoking, cholesterol, blood pressure and body mass index, BMI) (2) secondary prevention with aspirin, statin, beta-blocker; (3) cardiac investigation, including exercise ECG testing, thallium scanning, coronary angiography; (4) revascularisation with coronary artery bypass grafting (CABG) and/or percutaneous coronary intervention (PCI). Any previous history of myocardial infarction (MI) was identified. The number of face-to-face physician contacts in primary care over the previous 12 months was recorded. Data was also extracted concerning the current prescription of beta-blockers and statins (medication prescribed within the previous 6 months was considered to be current). Since aspirin can be purchased without a prescription over-the-counter, the 'advised, prescribed or previous' use of 'once daily aspirin' was noted. Contra-indications to aspirin use were also recorded.

### Statistical analysis

Odds ratios (OR) are used to summarise relative gender differences. An OR of 1.0 indicates no gender difference (greater than 1.0 favours men; less than 1.0 favours women). Adjusted male/female odds ratios (AOR) and their 95% confidence intervals were calculated using multiple logistic regression (SPSS v10). OR's were adjusted for current age, duration of angina (both in years) and previous MI. This also adjusted for age at diagnosis (current age minus angina duration). In a secondary analysis the annual number of physician contacts was included in the regression model.

The study complies with the Declaration of Helsinki. Ethical approval was obtained for the Liverpool Primary Care Data Project (the presence of clinical data managers in primary care sentinel practices with the collection and compilation of anonymised individual patient data) at its inception in 1992 from the appropriate Local Research Ethics Committees in Liverpool. Separate ethical approval was not required for the analysis of anonymised patient data reported in this study. Since the original ethical approval was only for the collection of anonymised individual patient data, it is not possible to link individual patient data with routinely available data concerning subsequent hospital admission or death certification.

## Results

The 8 sentinel practices had a combined registered population of 63,724 (31,977 aged 30 years or more). In the preceding 3 years 1,782 patients had received a nitrate prescription, of which 1,177 (66%) had been explicitly and unequivocally labelled as having angina pectoris (clinically certain angina). Their ages ranged from 32 to 95 years (94% were aged 50 years or more). The prevalence of nitrate treated angina was 3.7% (95%CI 3.5% to 3.9%) in those aged 30 years or more. 1,162 patients (610 men; 552 women) are included in this analysis (15 patients were excluded due to date of birth errors).

### Characteristics of patients with angina

There were important clinical differences between men and women with angina (see Table 1). Women were on average 3.6 (95%CI 2.3 to 4.8) years older than men; had a higher annual physician consultation rate (Mann-Whitney U test, p < 0.001); and had been 4.3 (95%CI 3.0 to 5.6) years older at diagnosis. Men had a longer duration of angina (a median of 6-months longer duration; Mann-Whitney U test, p = 0.01) and a higher prevalence of previous myocardial infarction (43% vs 25%; absolute difference 18.6%, 95%CI 13.2% to 23.9%).

**Table 1:** Characteristics of men and women with angina pectoris (N = 1,162)

	**Men**		**Women**	
	***n = 610***		***n = 552***	
Mean age, years (SD)	**66.9**	10.9	**70.5**	11.1
Mean age at diagnosis, years (SD)	**58.8**	10.9	**63.1**	11.8
Median duration of angina, years (IQR)	**6.9**	(3.5–11.4)	**6.3**	(2.8–10.6)
Median physician contacts 12 months (IQR)	**7**	(4–10)	**8**	(5–13)
Previous Myocardial Infarction, MI	265	**43%**	137	**25%**

SD, standard deviation; IQR, interquartile range.

### Overall clinical management of angina

**Figure 1:**
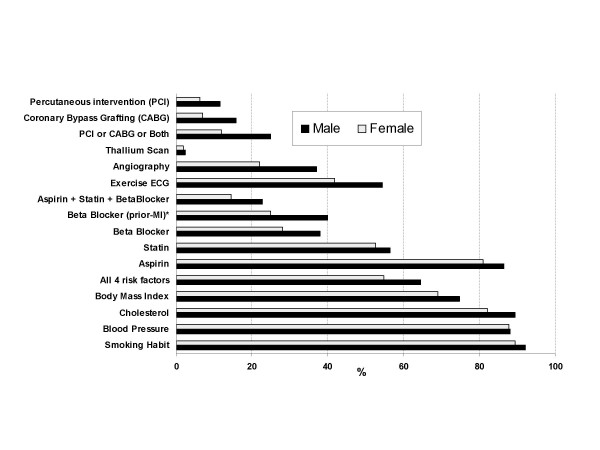
Bar chart of the clinical management of angina pectoris by gender (N = 1,162).

The clinical care provided to patients with angina is shown in Table 2 and Figure 1. The overall recording of cardiac risk factors was high. Smoking habit, blood pressure and cholesterol was recorded for more than 85% of patients, whilst BMI recording was somewhat lower (72%). Statins were prescribed to 55% of patients and 'once daily aspirin' was advised/prescribed to 84% of patients. Beta-blockers were currently prescribed to 35% of angina patients with a previous MI, which was similar to the use of beta-blocker by all patients (33%). Some 48% of patients with angina had undergone exercise ECG testing; 21% coronary angiography; and 19% revascularisation (9% PCI; 12% CABG)

**Table 2:** Gender differences in the clinical management of angina pectoris in primary care, N = 1,162 (a male:female odds ratio greater than one favours the clinical management of male angina)

	**Men**		**Women**		**Unadjusted**	Adjusted*	
	**(n = 610)**	**%**	**(n = 552)**	**%**	**Odds Ratio**	**Odds Ratio**	**95%CI**
**Risk Factor Recording**							
Smoking habit	562	**92**	493	**89**	1.40	**1.12**	0.74 to 1.71
Cholesterol	545	**89**	453	**82**	1.83	**1.46**	1.02 to 2.07
BP previous 12 months	537	**88**	484	**88**	1.03	**0.99**	0.69 to 1.43
Body Mass Index	456	**75**	381	**69**	1.33	**1.18**	0.90 to 1.54
All 4 risk factors recorded	393	**64**	302	**50**	1.50	**1.35**	1.06 to 1.73
**Secondary Prevention**							
Aspirin	527	**86**	447	**81**	1.49	**1.35**	0.97 to 1.86
Statin	344	**56**	290	**53**	1.17	**0.92**	0.72 to 1.18
Beta Blocker	231	**38**	155	**28**	1.56	**1.43**	1.10 to 1.86
Aspirin+Statin+Beta Blocker	138	**23**	80	**14**	1.73	**1.47**	1.07 to 2.02
Beta Blocker (prior MI, n = 402) **	106	**40**	34	**25**	2.02	**1.83**	1.12 to 3.00
**Investigation**							
Exercise Electrocardiograph	332	**54**	231	**42**	1.66	**1.31**	1.02 to 1.68
Coronary Angiography	228	**37**	123	**22**	2.08	**1.61**	1.23 to 2.12
Thallium Scan	15	**2.5**	10	**1.8**	1.37	**1.12**	0.48 to 2.63
**Revascularisation**							
PCI or CABG or Both	152	**25**	66	**12**	2.44	**1.93**	1.39 to 2.68
CABG	96	**16**	38	**7**	2.53	**2.02**	1.33 to 3.06
PCI	70	**11**	34	**6**	1.97	**1.59**	1.02 to 2.49

Adjusted odds ratios based on 1,161 complete cases (one patient lacked date of diagnosis for angina); 95%CI, 95% confidence interval; BP, blood pressure; PCI, percutaneous coronary intervention; CABG, coronary artery bypass grafting.

* Odds Ratios adjusted (AOR) for age, duration of angina and previous MI using multiple logistic regression.

** Previous Myocardial Infarction (MI), n = 402 (265 men; 137 women): odds ratio adjusted for age and duration of angina.

### Gender differences in angina management

Men received higher levels of care for their angina for almost all aspects of clinical management. Men were more likely to have their individual cardiac risk factors recorded (except for blood pressure measurement which was the same for both sexes) and were more likely to have all four risk factors recorded (Table 2). Men also received a higher level of secondary prevention therapy with aspirin, statins and beta-blockers. They were also more likely to have undergone further cardiac investigation and revascularisation. (Figure 1) The largest 'absolute' gender differences were in the performance of exercise ECG testing (12.6%, 95%CI 6.8% to 18.2%); coronary angiography (9.5%, 95%CI 4.9% to 14.1%), coronary revascularisation (13.0%, 95%CI 8.5% to 17.3%), and beta-blocker use by angina patients with a previous MI (absolute difference 15.1%, 95%CI 5.5% to 24.0%). The corresponding relative gender differences (odds ratios) are shown in Table 2.

### Adjusted comparison of gender differences

**Figure 2:**
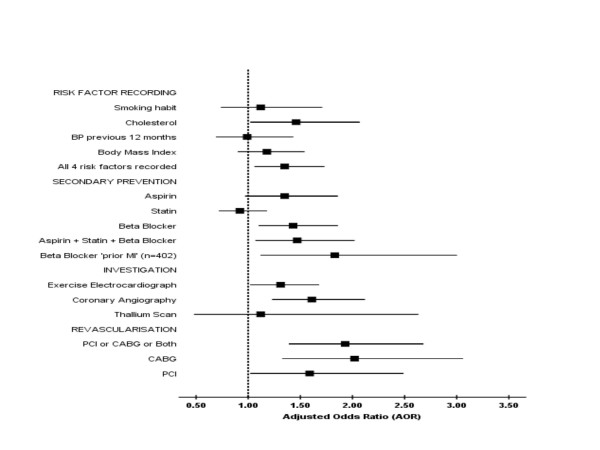
Forest plot of the clinical management of angina pectoris: male-to-female odds ratios adjusted for age, duration of angina and previous myocardial infarction (N = 1,162).

Duration of angina was unavailable for only one patient and consequently 1,161 patients were included in multiple logistic regression analysis (adjusted for age, angina duration and previous MI). All the adjusted odds ratios (AOR) were closer to unity than unadjusted OR's (Table 2). The AOR's are shown in Figure 2.

### Risk factor recording and secondary prevention

Men were significantly more likely to have all four risk factors recorded (AOR 1.35, 95%CI 1.06 to 1.73). The higher use of aspirin by men (AOR 1.35, 95%CI 0.97 to 1.86) was not accounted for by gender differences in the prevalence of contra-indications to aspirin (e.g. previous history of peptic ulceration/dyspepsia was similar for men and women; 11.7% v 12.9%). Men were also significantly more likely to be prescribed a beta-blocker, a gender gap that was wider for those with a previous MI (AOR 1.83, 95%CI 1.12 to 3.00). Men were also significantly more likely than women to be prescribed 'triple secondary prevention' with 'aspirin, statin and beta-blocker' (AOR 1.47, 95%CI 1.07 to 2.02). Statin use was the only aspect of clinical management that (non-significantly) favoured women (AOR 0.92, 0.72 to 1.18).

### Investigation and Revascularisation

Further cardiac investigation, with exercise-ECG testing and coronary angiography, was significantly higher for men (AOR 1.31 and 1.61 respectively). Thallium scanning was an uncommon investigation, but its limited clinical use also favoured men (AOR 1.12, not statistically significant). Men were significantly more likely than women to undergo revascularisation with both CABG and PCI (AOR 2.02 and 1.59 respectively). Overall the use of any of these cardiac investigations was significantly higher for men than for women (AOR 1.56, 95%CI 1.21 to 2.01).

### Physician contact

Including the annual number of primary care physician contacts in the regression model made no important difference to most of the AOR's reported in Table 2. For example, the AOR for aspirin use and revascularisation were 1.36 and 1.98 respectively (compared with 1.35 and 1.93 respectively). But adjusting for annual physician contact consistently widened the gender gap in relation to the more favourable recording of cardiac risk factors for men (Table 3). The largest changes occurred in relation to the recording of BP and smoking habit (AOR 1.29 and 1.43 respectively).

**Table 3:** Influence of physician contact on gender differences for cardiac risk factor recording in 1,161 patients with angina pectoris (odds ratio greater than one favours the recording of cardiac risk factors for men)

	**Adjusted Odds Ratios (95%CI)**	**Adjusted Odds Ratios (95%CI) also adjusted for annual physician contact**
	*N = 1,161*	*N = 1,155*
Body Mass Index	**1.18 **(0.90 to 1.54)	**1.27 **(0.96 to 1.67)
BP previous 12 months	**0.99 **(0.69 to 1.43)	**1.29 **(0.88 to 1.90)
Smoking habit	**1.12 **(0.74 to 1.71)	**1.43 **(0.92 to 2.22)
Cholesterol	**1.46 **(1.02 to 2.07)	**1.68 **(1.17 to 2.42)
All 4 risk factors recorded	**1.35 **(1.06 to 1.73)	**1.50 **(1.17 to 1.93)

BP, blood Pressure; 95%CI, 95% confidence interval.

All Odds Ratios are adjusted for: age, duration of angina and previous MI.

Odds ratios in the final column also adjusted for number of physician contacts over 12 months.

N, number of 'complete cases' included in regression analysis.

(number of physician contacts missing for 6 patients)

## Discussion

In this study, women with 'clinically certain angina' received significantly less favourable cardiac care than comparable men across a range of clinically important measures. Women were less likely to have their cardiac risk factor recorded, receive secondary prevention therapy, be referred for further cardiac investigation, or undergo revascularisation. These gender differences remained after adjusting for the older age of women with angina, their lower prevalence of a prior MI, longer duration of angina, and older age at diagnosis.

### Chronic stable angina

Only two previous studies have examined gender differences in the management of chronic stable angina in primary care and adjusted for important confounding factors such as age and previous MI [22,23]. We have reported a similar gender pattern in the management of angina in primary care 6 years previously, although a smaller sample size meant that only sex differences in the use of aspirin, exercise testing and revascularisation were statistically significant (AOR 2.07, 1.56 and 1.71 respectively) [22]. In our previous study women were also significantly less likely to be assessed by a cardiac specialist and were more likely to be managed entirely within primary care [22]. A larger Scottish study using routinely collected data has demonstrated a greater use of aspirin, statins and beta-blockers for men with angina, (AOR 1.21, 1.20 and 1.16 respectively), but did not assess risk factor recording, cardiac investigation or revascularisation [23]. Whilst a hospital-based study in England of the clinical investigation of angina found a greater use of exercise testing, angiography and revascularisation for men (AOR 2.65, 2.42, 2.80 respectively) [7].

### Coronary heart disease in primary care

Five UK studies have looked at specific aspects of CHD management in primary care and adjusted (at least) for age as a confounding factor [25-29]. Such studies have commonly used large prescribing databases without validating the diagnosis of CHD [25-28,30], or distinguishing between patients with angina, prior MI, or both [25-29]. Distinguishing between patients with 'angina alone', 'angina and prior MI' and 'prior MI-alone' is important, because hospital admission with an acute MI is likely to narrow any gender differences. Furthermore, gender differences in the clinical manifestation of CHD (as chronic stable angina or acute coronary syndrome) may also reflect underlying gender differences in the pathophysiology of atherosclerosis and plaque morphology [31-33].

In relation to CHD therapy, the use of statins is most commonly reported aspect of the gender differences observed in primary care [25-29,34]. Four studies have shown lower levels of statin therapy in women with CHD [25-28] and lower levels of cholesterol testing [25]. One study has also demonstrated the persistence of gender differences, for each and every year over a 6 year period (1997–2002), in the use of statins, aspirin, beta-blockers and ACE inhibitors [28]. By contrast the 1998 Health Survey for England found only minimal gender differences in the self-reported use of statins [34]. Another study (which verified the diagnoses of CHD against the medical records) also found no important gender differences in cardiac risk factor recording or the use of secondary prevention therapy such as statins [29]. This latter study also reported a higher level of revascularisation for men [29]. Whilst not based in primary care, the recent EuroHeart study (coordinated across 24 European countries) found a higher performance of exercise ECG and coronary angiography in men presenting to European cardiologists with stable angina [35].

### Study strengths and limitations

Our study has several important strengths. Firstly, experienced CDMs extracted the data according to an agreed protocol for a comprehensive range of measures that are relevant to the primary care management of angina [24]. In the UK, primary care medical records are a valuable source of information since they continuously collate data, over a prolonged period of time within the National Health Service (NHS), from both primary and secondary care. Unlike previous studies, we accessed and reviewed the complete primary care medical records to confirm that the patients in this study had a 'clinically certain' physician diagnosis of angina [24,30]. Our dataset does not allow us to assess the influence of physician gender on the management of angina. In the UK patients are often registered under one primary care physician (for administrative purposes), but actually consult a different physician within the practice/partnership.

Secondly, we identified a large number of patients from a diverse urban population receiving treatment for chronic stable angina pectoris. UK clinical guidelines advocate prescribing nitrates to all patients with 'clinically certain angina' and nitrate prescribing is a useful marker for chronic stable angina [24,30,36,37]. The prevalence of angina in our study is comparable with other studies, suggesting that we identified the majority of individuals being treated for clinically certain angina [37-39]. Thirdly, other than adjusting for previous MI we did not assess the severity of CHD, or the presence of important co-morbidities that might account for the differences that we found. Although previous research suggests that women's angina is often more disabling than men's [3,31,40,41], and that the sexes have similar levels of co-morbidity (including diabetes, hypertension and heart failure) [42]. An important omission in our study is that no data was collected concerning previous blood sugar measurements or the presence of diabetes. At the time of the study, UK guidelines advocated the measurement of body mass index (BMI) in the assessment of obesity. The measurement of renal dysfunction (estimated glomerular filtration rate) and waist/hip ratios in the routine assessment of patients with CHD has only recently been advocated in the UK.

Our study has some important limitations. Firstly, our sentinel practices were well-organised volunteers. Compared with the city of Liverpool as a whole they generally had smaller list sizes with more attached staff and younger physicians. Consequently they may provide a higher than usual standard of care. Previous studies have attempted to avoid such volunteer bias by utilising data from all GP practices in an urban area, but then compromised generalisability by excluding those practices unable to provide adequate data [29].

Secondly, our retrospective review of medical records relies inherently upon data recorded as part of routine clinical care. A prospective cohort study of incident cases of angina would be a much stronger study design, but would require considerably more resources. For example, the identification of 1,000 incident cases of angina would require the accurate surveillance of around 625,000 adults for 12 months [23].

Thirdly, other than adjusting for previous MI we did not assess the severity of CHD, nor the presence of important co-morbidities which might account for the differences that we found. Although previous research suggests that women's angina is often more disabling than men's [3,31,40,41], and that the sexes have similar levels of co-morbidity (including diabetes, hypertension and heart failure) [42]. An important omission in our study is that no data was collected concerning previous blood sugar measurements or the presence of diabetes. At the time of the study, UK guidelines advocated the measurement of body mass index (BMI) in the assessment of obesity. The measurement of renal dysfunction (estimated glomerular filtration rate) and waist/hip ratios in the routine assessment of patients with CHD has only recently been advocated in the UK.

Finally, our diagnosis of angina pectoris is based on subjective clinical judgement. Confirming the presence of CHD in primary care in the UK can be problematic, both in accessing cardiac investigations (such as exercise ECG or angiography) and interpreting the results [31]. Even in the absence of tests confirming cardiac ischaemia, 'nitrate treated angina' is strongly associated with a relative increase in the risk of death from CHD. A risk that is similar for both men and women [42]. When we restricted our analysis to the sub-group of patients with angina and a previous MI and (a group of patients who have clearly established the severity of their CHD) we observed the same clinical pattern of gender differences. Men were still significantly more likely to have their cardiac risk factors recorded, receive secondary prevention, and undergo further cardiac investigation and revascularisation. Although our confidence intervals were inevitably wider due to the smaller number of patients involved.

### Gender differences in healthcare

Our study has demonstrated that important systematic gender differences exist in the comprehensive clinical management of patients with angina in primary care. Our findings add support to the observation (in both the UK and USA) that when presented with simulated clinical vignettes, primary care physicians seem predisposed to adopt a less favourable approach towards the clinical management of women with cardiac symptoms compared to men [20,21]. There are several possible explanations as to why women may receive less favourable cardiovascular healthcare than men [17,19,31,35] and both biological and social factors are likely to be involved. Gender differences exist in clinical presentation of cardiovascular symptoms, with 'atypical' presentations being much commoner in women. The medical concept of 'classical angina' may disadvantage women since it is based upon the 'typical' male presentation of exertional chest pain. Diagnostic uncertainty is further exacerbated by a higher false-positive rate of exercise tolerance testing (ETT) in women. There are also widespread perceptions (both lay and professional) that women have a lower risk of developing CHD and a more favourable prognosis when they do. The willingness to offer and accept cardiovascular therapy and intervention may be influenced by the patients gender, whilst the technical challenge of operating on women's smaller coronary arteries may also augur against surgical intervention [17,19,31,35]. Wider social and cultural influences are also likely to play an important role, since gender differences in the provision of healthcare is not a phenomena that is solely restricted to CHD. Women also receive less favourable clinical care in relation to diabetes [43] and the performance of effective invasive procedures such a hip replacement and renal transplantation [44]. The first step in addressing the gender differences observed in the provision of CHD care is the wider appreciation, by both professionals and the public, of the existence of such differences along with a more accurate perception of the risk posed to women by CHD.

### Clinically appropriate gender differences

It has been suggested that gender differences in the management of CHD may be clinically appropriate given women's more atypical presentation, greater false positive rate on exercise EGC testing and smaller coronary arteries [31]. But in women in with clinically certain angina pectoris (explicitly and unequivocally labelled as having angina pectoris by a physician) it is difficult to accept the lower recording of cardiac risk factors and the lower use of effective secondary prevention therapy as being clinically appropriate. Major modifiable cardiac risk factors are equally likely to be found in men and women with angina [45] and there is no empirical evidence to suggest that secondary prevention therapy (with aspirin, statins, or beta-blockers) is less effective in women than in men [8].

### Changes in gender differences

This study was undertaken 18 months after the introduction of the National Service Framework for CHD in England [46], yet the pattern of CHD gender differences observed is largely unchanged from the one we observed 6 years earlier [22]. In relation to coronary revascularisation there has also been no appreciable narrowing of the gender gap in either the USA or the UK [5,6] and a recent Scottish study has reported a widening of the gender gap in the use of secondary prevention therapy [28].

## Conclusion

We have demonstrated that important gender differences exist in the primary care management of chronic stable angina in the UK. Women labelled with a clinical diagnosis of angina pectoris receive a lower level of risk factor assessment, secondary prevention therapy, cardiac investigation and coronary revascularisation. Such differences are not due to the lower prevalence of a prior MI among women, their older age or longer duration of angina. The relatively high level of risk factor assessment in women, although still below that for men, appears to be related to their higher primary care consultation rate.

## Competing interests

The author(s) declare that they have no competing interests.

## Authors' contributions

MC conceived the study. MC, LL & FJ designed the study. LL &FJ oversaw data collection. MC & XH undertook the analysis. MC, PB & XH critically interpreted the data. MC drafted the final manuscript. All authors critically read and approved initial drafts and the final manuscript.

## Pre-publication history

The pre-publication history for this paper can be accessed here:


